# Quality of life impairment in vitiligo: A comprehensive review of psychosocial and clinical determinants

**DOI:** 10.1177/03000605261444465

**Published:** 2026-05-04

**Authors:** Alice Ferreira Da Costa, Hasan Ashkanani, César Ferreira, Tiago Torres

**Affiliations:** 1 Instituto de Ciências Biomédicas Abel Salazar, University of Porto, Portugal; 2 Department of Dermatology, 532038 Al-Amiri Hospital, Kuwait; 3 Department of Dermatology, 26706 Centro Académico Clínico ICBAS/Santo António, University of Porto, Portugal

**Keywords:** Vitiligo, quality of life, health-related quality of life, psychosocial burden, stigma, depression, anxiety, dermatology

## Abstract

Vitiligo is a chronic depigmenting disorder that imposes a substantial psychological, social, and economic burden, extending beyond its physical manifestations. This narrative review synthesizes and critically evaluates current evidence on the determinants of quality of life impairment among individuals with vitiligo. A review of the published literature was conducted, focusing on studies evaluating health-related quality of life, psychosocial outcomes, stigma, psychiatric comorbidities, and validated quality of life assessment instruments in patients with vitiligo. Evidence from systematic reviews, cohort studies, cross-sectional analyses, and instrument validation studies was examined to identify the clinical, demographic, and sociocultural factors associated with disease burden. Across studies, vitiligo was consistently associated with significant impairment in psychological and social domains of quality of life. Disease visibility, particularly involvement of the face or genital regions; greater body surface area involvement; active disease progression; and longer disease duration emerged as key clinical factors associated with worse outcomes. Several demographic and sociocultural characteristics, including female sex, younger age, darker skin phototype, unmarried status, and residence in highly stigmatizing cultural environments, were also associated with greater quality of life impairment. Depression and anxiety were more frequently reported in individuals with vitiligo than in the general population, with pediatric and adolescent patients demonstrating particularly high vulnerability. Although dermatology-specific instruments such as the Dermatology Life Quality Index are commonly used in research and clinical practice, vitiligo-specific tools may more accurately capture stigma, social participation limitations, and disease-specific psychosocial impact. Overall, the available evidence indicates that vitiligo imposes a profound psychosocial burden that often exceeds the objective clinical severity of depigmentation. Quality of life impairment appears to be driven primarily by lesion visibility, sociocultural context, and psychological comorbidity rather than symptom severity alone. These findings underscore the importance of incorporating validated quality of life assessment tools into routine clinical care, screening for psychiatric comorbidities, and adopting culturally sensitive and patient-centered approaches to vitiligo management.

## Introduction

Vitiligo is an acquired, chronic inflammatory disorder characterized by the selective destruction of functional epidermal melanocytes, resulting in sharply demarcated depigmented macules and patches that vary in size and distribution.^1,2^ It affects both sexes equally and occurs across all skin phototypes, with a global estimated prevalence of 0.5%–2%.^2,3^ Although vitiligo can develop at any age, onset most commonly occurs early in life, with a substantial proportion of cases arising before the fourth decade.^1,2^ The disease is multifactorial, with genetic predisposition, autoimmune dysregulation, oxidative stress, neurogenic factors, and intrinsic melanocyte defects all implicated in its pathogenesis.^2,4,5^

Current consensus terminology classifies all nonsegmental variants under the broad term “vitiligo,” which includes generalized, acrofacial, mucosal, universal, and mixed forms. Generalized vitiligo is the most prevalent phenotype and is characterized by bilateral, often symmetrical lesions distributed across random body sites.^1,6^ Segmental vitiligo presents unilaterally, often following a dermatomal pattern, and demonstrates rapid progression during its early phase.^7^ Diagnosis is primarily clinical, particularly when lesions occur on sun-exposed areas such as the face and dorsal hands. Many patients report precipitating factors, including emotional stress and mechanical trauma, consistent with the Koebner phenomenon.^2,7,8^ Disease progression occurs in most cases, typically through the enlargement of existing lesions or appearance of new ones.^1,2^

Assessment of disease stability remains clinically important but is challenging to define. Although global scoring systems such as the Vitiligo European Task Force assessment aim to classify patients as progressive, stable, or regressive, individual lesions often evolve independently, thereby reducing the reliability of a single global metric.^9^ Serial lesion-specific monitoring, supported by patient history and digital imaging, is considered a more accurate approach for determining stability.^9^

Currently, no curative therapy has been established for vitiligo, and available treatments aim to halt disease progression and stimulate repigmentation.^10,11^ First-line therapies include narrow-band ultraviolet (UV) B phototherapy, topical corticosteroids, short-term systemic corticosteroids, topical calcineurin inhibitors, and topical ruxolitinib, which has been added recently. However, treatment responses remain highly variable.^12,13^ Surgical interventions, including grafting and melanocyte transplantation, may be considered for stable, therapy-resistant disease.^12^ Depigmentation therapies are reserved for select patients with extensive involvement, given their irreversible nature and significant psychosocial implications.^12^ New medical therapies, particularly oral Janus kinase (JAK) inhibitors, are emerging as highly promising options and may offer more consistent and durable repigmentation outcomes.^14^

Despite limited physical symptoms, vitiligo imposes a substantial psychosocial burden that often exceeds that associated with other inflammatory dermatoses.^15^ Visible lesions can profoundly affect self-image, social interactions, and occupational functioning, thereby contributing to elevated levels of stigma, anxiety, and depression.^16^ This burden is further amplified by the chronic nature of the disease, its unpredictable course, and the inconsistent efficacy of available treatments. For many individuals, the psychosocial consequences of vitiligo represent the most disabling aspect of the condition.

Growing recognition of these challenges has increased focus on evaluating quality of life (QoL) as an integral component of vitiligo management.^16^ QoL impairment reflects a complex interplay among clinical severity, lesion visibility, patient age, comorbidities, and sociocultural context.^16,17^ Understanding these factors and accurately assessing patient-reported outcomes is critical for guiding clinical decision-making and developing patient-centered care strategies.

This narrative review aimed to synthesize and critically evaluate the evidence on the psychological, social, and economic burden of vitiligo and to identify the clinical and demographic factors most strongly associated with QoL impairment.

This narrative review was conducted in accordance with the Scale for the Assessment of Narrative Review Articles (SANRA), which provides a structured framework for evaluating the methodological quality of narrative reviews. Relevant literature was identified through searches of PubMed, Scopus, and Google Scholar using combinations of the terms “vitiligo,” “quality of life,” “psychosocial burden,” “stigma,” “depression,” and “anxiety.” Systematic reviews, observational studies, and studies evaluating validated QoL instruments in patients with vitiligo were prioritized. Reference lists of relevant articles were also screened to identify additional studies.

## QoL in vitiligo: Definitions and assessment

### Defining QoL and HRQoL

QoL is a broad concept encompassing an individual’s overall well-being across various domains. The World Health Organization defines QoL as “individual’s perception of their position in life in the context of the culture and value systems in which they live and in relation to their goals, expectations, standards and concerns,” thereby highlighting its inherently subjective nature.^18^ In healthcare, the term health-related QoL (HRQoL) is used to denote the impact of health or disease on three key domains: physical, psychological, and social functioning.^19^ Physical functioning refers to the ability to carry out daily activities and the presence of any bodily symptoms or limitations; psychological functioning includes emotional well-being and cognitive health; and social functioning involves relationships and societal integration.^20^ Vitiligo itself does not typically cause physical pain or functional impairment of the body. However, its effect on appearance can indirectly impact the physical domain (e.g. avoiding activities such as swimming to hide lesions) and exert a substantial impact on psychological and social domains. The concept of QoL is multidimensional; vitiligo’s impact must be understood not just in terms of skin depigmentation but how that depigmentation affects a person’s mind, relationships, and daily life.^21,22^

### QoL impairment in vitiligo

Chronic skin diseases such as vitiligo can diminish overall well-being even in the absence of mortality or pain, as humans associate psychosocial importance with skin appearance.^23,24^ Patients with vitiligo seek treatment not for physical relief but to improve their appearance and self-esteem, regain confidence in social interactions, and reduce emotional distress. Studies have consistently shown that vitiligo is associated with significant QoL impairments in many life domains, including routine daily activities, clothing choices, work life, and interpersonal relationships. A systematic review on the humanistic burden of vitiligo found substantial negative effects on patients’ emotional health, social functioning, and overall mental health–related QoL.^5^ Visible lesions on the face, hands, or other prominent areas (or lesions in sensitive regions such as the genital area) substantially impair QoL compared with those in less noticeable locations.^25^ Moreover, vitiligo’s chronic, unpredictable course and the lack of a uniformly effective cure contribute to feelings of helplessness and frustration, thereby adding to the QoL burden.^5^ Recognizing these impacts, recent guidelines emphasize incorporating QoL assessment into vitiligo management.^5^

#### Assessing QoL in vitiligo: Tools and instruments

Because vitiligo’s burden is primarily psychosocial, patient-reported outcome measures are essential for quantifying its impact. Several instruments have been developed to measure QoL in dermatology, including tools specific to vitiligo. Table 1 summarizes common QoL assessment tools used in vitiligo. General health questionnaires (such as the Short Form-36 (SF-36)) are rarely used in vitiligo, as they may lack sensitivity to skin-specific concerns.^18,26^ Instead, dermatology-specific QoL scales (used across skin diseases) and vitiligo-specific scales are preferred for their sensitivity. The Dermatology Life Quality Index (DLQI) is one of the most widely used instruments. It comprises a 10-question survey covering six domains (symptoms/feelings, daily activities, leisure, work/school, personal relationships, and treatment), with scores ranging from 0 (no impact) to 30 (maximum impact).^27^ A DLQI score >10 indicates a substantial effect on a patient’s life.^28^ Notably, DLQI has been recommended by the European Academy of Dermatology and Venereology (EADV) as a standard QoL tool for vitiligo.^27^ However, because vitiligo is not associated with physical discomfort, patients often score lower on symptom-related DLQI items, potentially underestimating the true burden compared with diseases associated with symptoms of pain or itching.^28,29^ This limitation has driven the development of vitiligo-specific QoL scales that place greater emphasis on emotional and social impacts.^30^

**Table 1.** Common QoL assessment instruments used in vitiligo.

Instrument	Description/features
DLQI (Dermatology Life Quality Index)	Dermatology-specific 10-item survey covering six domains: symptoms/feelings, daily activities, leisure, work/school, personal relationships, and treatment. Total score 0–30; higher scores reflect greater impairment. Widely used in vitiligo and recommended by EADV.^20^ May underestimate vitiligo’s burden because physical symptoms are minimal.^22^
VitiQoL (Vitiligo Quality of Life questionnaire)	Vitiligo-specific instrument with 16 items across three domains: stigma, participation limitation, and behavior.^24^ Captures psychosocial effects such as shame, avoidance, and coping behaviors. Validated for adults and sensitive to clinical change.
VIS (Vitiligo Impact Scale)	Vitiligo-specific scale developed in India, with emphasis on social stigma and cultural attitudes toward vitiligo.^26^ Particularly useful for stigma-related distress; performance may vary across cultural settings.
VLQI (Vitiligo Life Quality Index)	Vitiligo-specific index with a 1-week recall period, enabling detection of short-term QoL changes, such as after treatment.^27^ Focuses on emotional and daily-life impact, with little emphasis on physical symptoms.
VIPs (Vitiligo Impact Patient scale)	The first vitiligo-specific burden tool; includes versions for fair skin (phototypes I–III) and dark skin (IV–VI).^28^ Assesses multiple domains of disease burden, accommodating differences in visibility and perceived stigma based on skin type.
CDLQI (Children’s Dermatology Life Quality Index)	Dermatology-specific questionnaire for ages 4–16, with 10 items addressing school, friendships, play, teasing, sleep, and treatment.^29^ Commonly used to assess QoL in pediatric and adolescent vitiligo.

QoL: quality of life; EADV: European Academy of Dermatology and Venereology.

#### Vitiligo-specific QoL instruments

Several scales have been designed particularly for vitiligo (Table 1). For example, the Vitiligo QoL questionnaire (VitiQoL) is a validated tool comprising 16 items across three domains (stigma, participation limitation, and behavior), specifically tailored to patients with vitiligo.^31^ Additional tools include the Vitiligo Impact Scale (VIS), Vitiligo Life Quality Index (VLQI), and Vitiligo Impact Patient scale (VIPs).^32^ These instruments often capture aspects such as stigma and camouflaging behavior that may be not be addressed using general dermatology surveys. A study reported that VIS, developed in India, specifically addresses cultural stigmatization issues; however, its performance may vary across different populations.^33^ The VLQI uses a short 1-week recall period, making it sensitive to changes over brief intervals (such as before and after a treatment trial).^34^ VIPs was among the first vitiligo-specific scales that uniquely provides versions for different skin phototypes (to account for the varying contrast and sociocultural impact in fair vs. dark skin).^35^ For pediatric patients, the Children’s Dermatology Life Quality Index (CDLQI) is commonly used (for ages 4–16), as recommended by guidelines.^36^ Each tool has its advantages and disadvantages, and currently, no single vitiligo-specific QoL instrument is fully validated or universally adopted.^37^ Although DLQI remains the most used measure in studies and clinical practice,^27^ there is an urgent need for a robust, validated vitiligo-specific QoL instrument that is applicable across various ages, sexes, and cultures.^38^

Selecting an appropriate QoL tool is important in both research and clinical practice. In vitiligo clinical trials, QoL outcomes (often DLQI or VitiQoL) are increasingly reported alongside repigmentation findings.^38,39^ In routine practice, use of a short questionnaire can help clinicians identify patients’ hidden distress. Regular QoL assessment is recommended to identify and promptly address significant psychosocial suffering.^40,41^

### Clinical factors affecting QoL

Although the impact of vitiligo on QoL impact is largely attributed to visible appearance changes, certain clinical aspects of the disease can modulate the degree of impairment. Key disease-related factors include the location and extent of lesions, disease activity/progression, and any associated symptoms or comorbidities. These factors influence how noticeable the condition is and how challenging it is to manage, thereby affecting patient well-being.

#### Lesion distribution and visibility

The most critical determinant of QoL in vitiligo is whether lesions are located in highly visible areas. Lesions on the face, neck, arms or hands, and legs, areas not easily covered by clothing, are associated with significantly greater self-consciousness and perceived stigma.^42^ In a large survey, patients with vitiligo on visible body parts reported feeling more embarrassed and socially anxious, and they rated their disease as more severe.^43^ Visible lesions often draw unwanted attention or questions from others, leading to feelings of shame and avoidance of social interactions. For example, facial lesions were identified as the strongest driver of patient-perceived disease severity in a study.^43^ Lesions in certain sensitive areas also disproportionately affect the QoL. Vitiligo affecting the genital region can lead to sexual dysfunction or intimacy problems (discussed later), and lesions on the scalp or around body orifices may be difficult to hide.^43,44^ In contrast, lesions in areas normally covered by clothing may be less psychologically burdensome for some patients (although not all, as patients are often aware of their presence).

#### Extent of depigmentation

The total body surface area (BSA) affected by vitiligo is another important factor. Studies consistently demonstrate that patients with more widespread vitiligo report worse QoL.^45–47^ For instance, involvement of >5% affected body area has been associated with significantly higher vitiligo-specific QoL scores (indicating greater impairment).^48^ A French study found that involvement of >5% BSA, particularly when lesions were in visible regions, resulted in markedly higher QoL impairment.^49^ Some researchers have proposed thresholds such as >25% BSA as a marker of severe disease burden.^50^ Patients with extensive vitiligo face more challenges in concealing lesions and may feel a greater loss of control, potentially diminishing hope of regaining normal pigmentation.^47^ This can erode optimism about treatment outcomes and is associated with feelings of hopelessness.^47^ Conversely, very limited vitiligo (small patches) may have a milder impact; nevertheless, even a tiny lesion on a highly visible spot (e.g. the tip of the nose) can be distressing.

#### Disease activity and duration

Vitiligo is typically a chronic condition with an unpredictable course of spread, stabilization, and occasional spontaneous repigmentation or relapse.^51^ Actively spreading vitiligo tends to cause more anxiety and QoL decline compared with stable disease. Patients often report that the appearance of new white patches is one of the most distressing experiences, even when patches at other locations are repigmenting.^52^ A study reported that patients with actively spreading vitiligo exhibited significantly worse DLQI scores than those with a stable vitiligo.^53^ Although the duration of disease also plays a role, findings are inconsistent. Longer disease duration (many years) can worsen QoL due to cumulative frustration.^54^ Some studies have found that QoL is lowest in patients with a disease duration of 5–15 years, possibly reflecting weariness with chronic management or disappointment with treatment outcomes.^54^ Interestingly, patients with vitiligo for <5 years or conversely >20 years sometimes report better QoL than those in the mid-range,^55^ owing to optimism regarding the treatment or adaptation to their condition, respectively.^56^ Nonetheless, prolonged vitiligo is also associated with extended exposure to social stigma and emotional strain, which often contributes to ongoing psychosocial burden.^57^

#### Symptomatology

Traditionally, vitiligo is described as asymptomatic (no itching or pain), and many patients do not experience physical discomfort. However, a subset of patients report pruritus (itching) or a burning sensation in some lesions, which can contribute to distress. A study found that patients who had itching in their vitiligo patches also reported worse QoL, possibly because the symptom was bothersome and may indicate active inflammation. Proposed mechanisms for itch in vitiligo include local inflammatory mediators or heightened sensitivity of depigmented skin.^58^ Additionally, vitiligo lesions lack melanin, making the affected skin more prone to sunburn. This photosensitivity forces patients to strictly avoid sun exposure or use high–sun protection factor (SPF) sunscreen and protective clothing.^59^ Patients often modify their lifestyles (avoiding outdoor activities and swimming, among others) due to fear of sunburn or skin cancer in depigmented areas.^60^ Such adjustments, although medically protective, can restrict leisure activities and further remind patients of their condition, thereby affecting QoL.^61^ Fear of UV exposure and constant vigilance about sun protection contribute to an added daily burden for many, especially in sunny climates.^60^ Therefore, although vitiligo is not physically painful, any accompanying symptoms or necessary behavioral precautions can heighten the overall burden on QoL.

#### Autoimmune and other comorbidities

Vitiligo has been associated with other autoimmune diseases (e.g. thyroiditis, type 1 diabetes, alopecia areata, and psoriasis) in approximately 10%–20% of patients,^62^ and these comorbidities can exacerbate QoL impairment. For example, autoimmune thyroid disease not only requires additional management but also increases stress and health-related concerns in a patient with vitiligo. A nationwide study in France (VIOLIN cohort) found that patients with vitiligo with at least one concomitant autoimmune disorder had more extensive skin involvement and worse QoL scores.^63^ Some comorbidities (such as alopecia areata) are themselves visible and can amplify concerns related to appearance and self-esteem. The presence of multiple conditions also require more frequent doctor visits and treatments, contributing to financial and time burdens (addressed in the Economic Impact section). It is important for clinicians to screen patients with vitiligo for common comorbidities, as managing those effectively may help alleviate overall QoL burden.^64^ Conversely, psychiatric comorbidities (such as depression or anxiety disorders) are common and can be considered both outcomes and drivers of worse QoL; these will be discussed in the Psychological Impact section.

In summary, clinical characteristics such as widespread, highly visible, actively spreading vitiligo and co-occurring health issues tend to worsen the QoL impact.^64^ Patients with hard-to-hide or rapidly changing vitiligo often experience greater psychological distress and social avoidance. Recognizing these high-risk clinical scenarios is important for prompt intervention with appropriate counseling, more aggressive treatment (when desired by the patient), and support by healthcare providers.

Overall, vitiligo severity, visibility, and disease activity are the strongest clinical drivers of QoL impairment, with facial, genital, and extensive disease producing the greatest psychosocial burden. The main predictors of poor quality of life in vitiligo are summarized in Table 2.

**Table 2.** Major predictors of poor quality of life in vitiligo.

Domain	Predictor
Clinical	Facial/genital lesions, >5% BSA, active disease
Demographic	Female sex, younger age, darker skin
Cultural	High-stigma societies
Psychological	Depression, anxiety, and low self-esteem
Social	Lack of partner or family support

BSA: body surface area.

### Sociodemographic and cultural factors

In addition to clinical features, various patient-related factors influence the severity of vitiligo on QoL. Age, sex, skin tone, cultural context, and marital status are some key variables that have been studied.

#### Sex

Women with vitiligo generally report greater QoL impairment than men.^64^ This sex disparity is consistently observed across several studies. Societal pressures regarding appearance tend to be higher for women, which may explain why women often feel the impact more acutely.^2^ Female patients commonly report lower self-esteem, higher rates of anxiety or depression, and more social withdrawal than male patients.^65^ In many cultures, women feel compelled to conceal vitiligo patches with clothing or makeup, and the effort of constant camouflage can be burdensome. Overall, female patients appear to carry a heavier psychosocial burden of vitiligo, including more frequent experiences of shame, social embarrassment, and even sexual dysfunction (as discussed later).

#### Age

Age can influence QoL outcomes in vitiligo. Generally, younger people (especially adolescents and those in their 20s) experience a greater negative impact than older patients.^2^ Adolescence and young adulthood are life stages when social acceptance, body image, and peer perception are particularly important. Vitiligo during these years can severely disrupt self-image, leading to heightened self-consciousness and stress in social situations. Multiple studies have found that patients approximately <30 years exhibit lower QoL scores (indicating worse QoL) than those >50 years.^15^ Younger patients more often report depression, anxiety, or embarrassment associated with vitiligo.^15^ With increasing age, some patients appear to adapt or feel less social pressure regarding appearance, which may improve adjustment. However, the relationship is not strictly linear. Some older patients with long-standing vitiligo continue to exhibit significant burden, possibly due to cumulative frustration or more extensive disease (as vitiligo often progresses with time).^57^ However, middle-aged and older individuals may have certain concerns (such as marriage prospects), potentially mitigating some social impact. Therefore, older individuals with very extensive vitiligo or those who have faced prolonged stigma can report poor QoL.

#### Skin phototype and ethnicity

The contrast between vitiligo patches and one’s normal skin tone influences how noticeable the condition is. People with darker skin phototypes often report greater QoL impairment caused by vitiligo than those with very fair skin.^2^ In deeper skin tones, the stark visibility of white patches may attract more stares or probes. Moreover, cultural factors intertwine with this; in certain regions (e.g. South Asia, the Middle East, and parts of Africa), vitiligo is highly stigmatized, and studies from these regions often show higher average QoL burden than similar studies in Europe.^3^ For example, studies from India, the Middle East, and Turkey consistently demonstrate a larger QoL burden than those from Western Europe.^3^ Higher mean DLQI scores were reported in patients with vitiligo from Turkey and India than in Italian patients, reflecting both the greater visibility on darker skin and cultural attitudes toward appearance.^3^ Nevertheless, some African-American patients with vitiligo also face unique challenges, as the contrast can be extreme (jet-black skin against chalk-white patches) and lack of awareness in some communities can contribute to misunderstanding or fear. Meanwhile, individuals with very fair skin (Fitzpatrick I/II) can sometimes camouflage patches more easily (especially when they avoid tanning), which may reduce the social impact in certain cases. However, even patients with fair skin can be affected. Patients with lighter skin were found to be worried more about skin cancer risk in vitiligo, whereas patients with a darker skin were more concerned about disfigurement,^3^ indicating different concerns across groups, all of which are relevant QoL issues. Overall, ethnic and cultural context plays an important role. In settings where lighter skin is idealized (in some cultures fair skin is a beauty standard), vitiligo on dark skin may be particularly stigmatizing and vice versa. The key point is that the visibility of vitiligo, whether due to skin contrast or cultural scrutiny, drives much of its psychosocial impact.^3^

To further illustrate these differences, a comparative overview of vitiligo impact by skin phototype is presented in Table 3.

**Table 3.** Differential impact of vitiligo by skin phototype.

Feature	Light skin phototypes (Fitzpatrick I to III)	Dark skin phototypes (Fitzpatrick IV to VI)
Visual contrast of lesions	Low to moderate	High to extreme
Detectability in public	Often subtle, especially if untanned	Immediately visible
Social attention	Often unnoticed	Frequent staring and questioning
Stigma risk	Moderate	High
Cultural misinterpretation	Rare	Common in Asia, Africa, and Middle East
Marriage and relationship impact	Variable	Frequently severe
DLQI/VitiQoL scores	Lower on average	Higher on average
Camouflage effectiveness	Often effective	Often limited
Psychological burden	Moderate	High
Internalized stigma	Lower	Higher

DLQI: Dermatology Life Quality Index; VitiQoL: Vitiligo quality of life questionnaire.

#### Marital status and social support

Studies suggest that unmarried patients may experience a higher psychological burden from vitiligo than those who are married.^60^ This may be because single individuals are often actively seeking partners and may fear rejection due to their appearance. In cultures where arranged marriages are common, vitiligo can unfortunately reduce one’s marriage prospects. There are reports of vitiligo being considered a reason for rejection of a potential bride or groom in certain communities.^60^ Women with vitiligo, in particular, have faced difficulties finding spouses in traditional societies, and in some cases, the onset of vitiligo after marriage has been associated with a higher risk of divorce, owing to stigma or misconceptions.^60^ Conversely, some studies have reported that married patients often have had longer disease duration and possibly developed coping strategies with spousal support. For example, a study found that longer marriage duration was associated with better QoL in patients with vitiligo, suggesting that support and acceptance from a long-term partner can buffer psychosocial stress.^49^ In general, social support (from family, friends, or patient groups) is a protective factor. Patients who feel supported tend to report lower levels of depression and better adaptation.^51^ Therefore, isolation, whether due to being single or lack of social support, can worsen the perceived burden, whereas understanding from loved ones can help mitigate it.

#### Cultural and religious beliefs

Culture profoundly shapes the experience of vitiligo. In some regions, vitiligo is surrounded by misconceptions; for instance, in some cultures, it is believed that it is contagious or caused by past sins or bad karma.^60^ In India, derogatory terms for vitiligo and conflation with leprosy have been documented, resulting in severe social ostracization of patients.^62^ Studies from the Indian subcontinent report that patients with vitiligo may be barred from certain community activities, have trouble finding employment, and are stigmatized in social rituals or festivals.^60^ Similar stigma exists in parts of the Middle East and Africa, where vitiligo may be regarded as a curse or divine punishment. These cultural stigmas intensify the social impact. Patients internalize shame and may withdraw from public life altogether to avoid scorn.^60^ By contrast, in Western societies, awareness of vitiligo has improved somewhat in recent years (helped by public figures with vitiligo, e.g. model Winnie Harlow, openly embracing their skin). Nevertheless, even in the West, emphasis on physical perfection (reinforced by media and beauty standards) can make any visible difference, such as vitiligo, a source of self-consciousness.^60^ A concept known as internalized stigma occurs when patients start believing negative societal attitudes and feel inferior or unworthy. This phenomenon has been documented in individuals with vitiligo, particularly among those with low self-esteem.^61^ Religion can also play a role; for instance, in some belief systems, vitiligo is associated with impurity or divine displeasure. Therefore, it is crucial to understand a patient’s cultural background when evaluating their QoL. Even simple steps such as clarifying that vitiligo is not infectious or caused by diet can help dispel myths in the patient’s family and community, thereby reducing blame and fear.^44,61^ Culturally sensitive education campaigns have been suggested to combat vitiligo stigma in high-prevalence regions.^61^

Large population-based and hospital-based studies from India, China, and sub-Saharan Africa consistently demonstrate that vitiligo is associated with disproportionately severe stigma, marriage exclusion, and social avoidance compared with Western cohorts. In India, VIS was originally developed by Parsad et al. to capture culturally specific stigma, including marriage rejection and social ostracization.^50,66^ Chinese cohort studies report high rates of concealment behavior, anxiety, and family-related distress.^67,68^ African studies document widespread misbeliefs associating vitiligo with contagion, curses, or divine punishment, resulting in school exclusion, workplace discrimination, and marital instability.^69,70^

Therefore, both individual patient factors and societal perceptions significantly influence the impact of vitiligo. A young, single woman with vitiligo in a society with strong colorism and marriage stigma is likely to experience a substantially greater burden than an older, married man in a more accepting environment. Recognizing these sociodemographic and cultural factors is essential for comprehensive care. For example, a teenage girl may benefit from counseling and connecting with peer support to build resilience against bullying, whereas an adult in a community with widespread misconceptions may require educational resources for their family or community to reduce stigma.

The global sociocultural burden of vitiligo across different regions is summarized in Table 4.

**Table 4.** Global sociocultural burden of vitiligo.

Region	Key stigma features	QoL impact
India	Leprosy mislabeling and marriage rejection	Very high
China	Family shame, concealment, and anxiety	High
Africa	Curse and contagion beliefs	Very high
Middle East	Marriage and social stigma	High
Europe/North America	Appearance-based distress	Moderate

QoL: quality of life.

### Psychological impact

The impact of vitiligo on mental health and psychological well-being is one of the most documented aspects of the condition, with QoL substantially affected. The sudden or progressive loss of skin color, particularly at a young age, can be traumatic. Patients report a range of emotional responses, including shock, sadness, anger, embarrassment, and fear about the future.^58^ Unlike some medical illnesses that remain “invisible,” vitiligo alters the individuals’ appearance, affecting their identity and self-image. Herein, we examined the psychological burden in terms of self-esteem, emotional distress, and psychiatric comorbidities such as depression and anxiety.

Depression, anxiety, and low self-esteem are not secondary features but core components of vitiligo morbidity and should be routinely screened and managed.

#### Self-Esteem, body image, and emotional distress

The appearance of vitiligo patches frequently causes patients to become self-conscious. Many report feeling less attractive or even “disfigured,” which can contribute to low self-esteem and feeling of shame. Patients commonly attempt to hide their patches with clothing or makeup, which reflects the self-image challenges they experience.^36,58^ Qualitative interviews of patients with vitiligo reveal that they often avoid mirrors, photographs, or well-lit environments because watching their reflection or having others see their skin can trigger distress. There is a constant psychological strain of worrying about how others perceive them. Young adults with vitiligo, in particular, report feeling “different” and less confident compared with their peers, sometimes leading to social withdrawal to avoid judgment.^58^ This loss of confidence can also extend to professional settings; patients may hesitate in job interviews or public-facing roles owing to their appearance. Chronic embarrassment and stigmatization can, over time, foster feelings of worthlessness or hopelessness. A large systematic review reported that patients with vitiligo frequently experience stigmatization and feelings of social rejection, which associate with poorer overall psychological well-being.^57^ Internalizing stigma (believing negative stereotypes about oneself) further damages self-worth.^62^ Encouragingly, participation in vitiligo support communities or seeing role models with vitiligo can help counteract these negative self-perceptions by normalizing the condition and boosting self-acceptance. Nevertheless, emotional distress, ranging from frustration and sadness to more severe forms such as clinical depression, is common when vitiligo significantly alters a person’s body image.^71^ Nonetheless, not every patient is devastated psychologically; some cope well or even embrace their uniqueness. However, a considerable proportion struggle with the emotional burden, especially in the absence of support.

#### Depression and anxiety

Numerous studies have reported a higher prevalence of depressive and anxiety disorders among individuals with vitiligo than in the general population.^18,72^ Meta-analyses have quantified this burden. For example, Lai et al.^60^ found that approximately one-third of patients with vitiligo exhibit at least mild depressive symptoms, whereas approximately a quarter meet the criterion for major depression. Similarly, a 2021 systematic review by Ezzedine et al. reported very broad ranges for prevalence, with some studies showing as high as approximately 60% of patients experiencing depression or anxiety; however, a significant proportion of patients were affected.^17^ In this review (which pooled data from 168 studies), depression was the most commonly reported psychosocial comorbidity, found in 0.1%–62% of patients across studies (reflecting wide variability in populations and measurement).^18^ Anxiety disorders were also frequently reported (in approximately 1.9%–68% of patients).^18^ Although exact percentages may vary, the consensus is clear: patients with vitiligo are at higher risk of clinical depression and anxiety than people without vitiligo.^72^ These mental health conditions arise from the chronic stress of living with a visible difference and from frequent experiences of social avoidance or ridicule. Patients may develop social anxiety, fearing scrutiny in public settings. They may also develop generalized anxiety or persistent low mood from constantly worrying about disease progression and its potential impact on life milestones. Importantly, the presence of clinical depression or anxiety in vitiligo is associated with specific patient subgroups. Female, younger, single, or who have lesions in highly visible or stigmatizing locations (face, hands, or genital areas) are at a greater risk of developing psychological comorbidities.^18,57^ Greater disease extent and longer duration (≥5 years) are also associated with higher rates of depression.^18^ For instance, some studies have reported that patients with vitiligo in regions with high stigma (e.g. parts of Asia or Africa) have markedly higher rates of depression than those in less stigmatizing environments, reflecting the influence of social context and skin contrast on psychological impact. These patterns are consistent with earlier observations that individuals under greater social pressure (by virtue of demographics or disease severity) bear a heavier psychological burden.

From a clinician’s perspective, these findings underscore the need to screen patients with vitiligo for depression and anxiety. Simple tools such as the Patient Health Questionnaire-9 (PHQ-9) (for depression) or Generalized Anxiety Disorder-7 (GAD-7) (for anxiety) can be used in dermatology clinics when a patient shows signs of distress. Addressing these comorbidities is essential not only for mental health but also for the skin condition. Evidence suggests a bidirectional link, where psychological stress can aggravate vitiligo progression. Severe stress is often reported as a triggering or exacerbating factor in vitiligo onset.^61^ The hypothesized mechanism is that stress triggers a neuroendocrine cascade (e.g. catecholamine release and oxidative stress) that can adversely affect melanocytes.^61^ Additionally, depression may worsen vitiligo by promoting neglect of self-care or treatment nonadherence. Conversely, managing the skin condition (e.g. achieving repigmentation) can sometimes improve mood. Therefore, vitiligo and psychological health are intertwined in a cycle: vitiligo contributes to distress, and distress, in turn, can aggravate vitiligo’s activity.^61^ Breaking this cycle via psychological or psychiatric interventions can be a key component of comprehensive vitiligo care.

#### Psychological impact in pediatric patients

Special attention must be given to children and adolescents with vitiligo, as they are in formative stages of emotional development. When vitiligo appears in childhood, it can have long-term effects on personality, social development, and self-esteem.^63,64^ Studies of pediatric vitiligo show that even young children (as young as 5 or 6 years) are aware of looking different. By adolescence, the psychosocial impact can be as high or higher than that in adults. A survey of patients >18 years found that >90% of adolescents (ages 15–17 years) were bothered by their condition, compared with approximately half of younger children.^51^ This indicates that self-consciousness intensifies with age and puberty. Many adolescents with vitiligo experience teasing or bullying at school. For example, a German study reported that approximately half of children with vitiligo had received insulting comments and 22% had been bullied because of their skin.^63^ Such negative experiences at school can lead to school avoidance, decline in academic performance, and withdrawal from peer activities. Children with vitiligo may become more shy, anxious, or depressed. Adolescence, in particular, is a vulnerable period when identity is being formed and peer acceptance is paramount. When vitiligo develops around the preteen or teen years, it often has a considerable impact on self-image.^51^ Silverberg & Silverberg reported that the onset of vitiligo after 10 years (around puberty) of age resulted in worse QoL in adolescents than onset in early childhood.^49^ They hypothesized that a teenager who suddenly develops vitiligo experiences a significant disruption at a time when appearance is becoming central to identity. In contrast, a child who grew up with vitiligo from the toddler years may have somewhat adapted by their teens (although they still face challenges). Common issues reported by youth with vitiligo include reluctance to participate in sports (especially swimming or activities that expose skin), difficulty making friends, and fear of future relationships.^64^ Parents of children with vitiligo also experience distress. Many parents feel guilt or sadness regarding their child’s condition, and some develop anxiety or depression themselves.^16^ In a survey, 26% of parents of a child with vitiligo reported depressive symptoms and 42% had significant anxiety.^16^ This “secondary” psychological impact on families highlights vitiligo’s ripple effect. Encouragingly, children can be resilient with proper support. Early interventions, including counseling for the child (and family), connecting them with others who have vitiligo (so they do not feel alone), and educating school staff and peers regarding vitiligo, can considerably help reduce bullying and improve the child’s coping skills.^44^ Camouflage therapies (makeup) are even used in some children, with reports of improved confidence after learning to cover lesions.^36^ Overall, addressing the psychological needs of pediatric patients with vitiligo is crucial to prevent long-term self-esteem issues.

In summary, the psychological impact of vitiligo is profound and multifaceted. Depression, anxiety, low self-esteem, and even disordered behaviors (such as social phobia or adjustment disorders) are well-documented in a substantial proportion of patients.^18^ This reinforces that vitiligo management must extend beyond treating the skin to include mental health screening and support. Dermatologists are increasingly aware of this, and some clinics now work closely with mental health professionals to help patients cope with the emotional burden. Simple steps such as acknowledging the patient’s feelings, providing reassurance that those feelings are common and valid, and encouraging participation in support networks (in-person or online) can make a substantial difference.^44^ Ultimately, improving the QoL of a patient with vitiligo often requires treating the mind as much as treating the skin.

### Social impact

The social consequences of vitiligo are closely intertwined with psychological effects but warrant separate discussion. Social impact refers to how the condition affects a person’s interactions, relationships, role in society, and experiences of stigma or discrimination. Vitiligo is a visible difference that society may not always understand, and such lack of awareness can foster prejudice. Patients commonly report altered social behavior: some withdraw from gatherings, others avoid dating or become introverted, and many face direct stigma in their communities.^58^ We have examined key aspects of vitiligo’s social impact, including stigma, relationships (friendships, romantic, and marital), and occupational/academic implications.

Stigma, relationship disruption, and occupational discrimination represent major but modifiable drivers of vitiligo-related disability.

#### Stigma and discrimination

Stigma is a powerful social force defined as a mark of shame or discredit that sets a person apart. In visible dermatological diseases such as vitiligo, stigma arises when others notice the difference and attribute negative stereotypes to it (e.g. assuming it is contagious, caused by poor hygiene, or a “divine punishment”).^62^ Germain et al. described stigma in skin diseases as a multistep process. First, a difference is noticed and labeled; second, it is associated with stereotypes; third, a separation of “us” versus “them” occurs; and finally, this leads to status loss and discrimination against the affected person.^59^ This conceptual model fits vitiligo well. Historically, in some cultures, vitiligo was even colloquially known as “white leprosy.” Even today, studies report troubling statistics. For example, in India, a significant fraction of the general public holds false beliefs that vitiligo is infectious or caused by past misdeeds.^62^

#### Internalized stigma and social avoidance

Many patients begin to self-stigmatize, meaning they anticipate stigma and, as a result, alter their own behavior. They may isolate themselves to avoid the pain of being ostracized. Qualitative accounts suggest that patients skip social events (parties, gyms, and swimming pools) because they feel embarrassed or fear negative reactions.^58^ Some even avoid making new friends or entering new environments due to fear of being judged. This self-imposed withdrawal can unfortunately reinforce feelings of loneliness and depression. Importantly, not all social reactions to vitiligo are negative. Many people respond with curiosity or empathy. However, a patient who have experienced a few hurtful incidents may begin to expect negative responses in most social situation. Therefore, addressing stigma requires both societal education and patient counseling to rebuild social confidence.

On a hopeful note, public awareness of vitiligo has improved in recent years. Campaigns and representation (e.g. models or actors with vitiligo in media) help normalize the condition. The success of a high-profile model with vitiligo, for instance, has been cited by some patients as helping their self-image and reducing public ignorance. Nevertheless, further research is warranted in many parts of the world to reduce stigmatizing attitudes.

#### Relationships and intimacy

Vitiligo can significantly affect interpersonal relationships. With friends and family, patients sometimes feel the need to explain their condition or even conceal it around certain relatives. The support of close friends and family often determines how well a person copes socially, and individuals with understanding families tend to fare better. However, in the context of romantic relationships and sexual intimacy, vitiligo’s impact can be particularly challenging. Many patients express fear of rejection by potential partners upon noticing their patches, leading to avoidance of dating or intimate situations. A systematic review reported that >25% of patients with vitiligo experience some degree of sexual dysfunction attributable to their condition.^16^ Female patients, in particular, reported feeling undesirable or worried about disrobing in front of a partner.^64^ Studies that specifically assessed sexual QoL (using validated sexual function questionnaires) have revealed notable findings. Women with vitiligo have higher rates of problems with sexual arousal, satisfaction, and frequency of intercourse compared with women without vitiligo.^2^ In men, vitiligo appears to less commonly affect sexual function directly; however, some report reduced sexual satisfaction, potentially related to self-image concerns.^64^ Genital involvement of vitiligo is an important factor. Women with genital vitiligo had significantly higher sexual dysfunction scores than those without genital lesions in a study.^64^ However, even lesions on nongenital but highly visible areas can indirectly negatively affect intimacy due to overall body image concerns. A 2023 review by Liang et al. concluded that patients with vitiligo have a higher risk of sexual dysfunction, particularly among females, largely due to psychological factors such as shyness and low self-esteem in intimate settings.^63^ Essentially, when a patient is anxious or ashamed of their skin, it is difficult for them to feel comfortable during intimacy, affecting sexual desire and satisfaction.^64^

In contrast, evidence suggests that when vitiligo is well-understood and accepted by one’s partner, the negative impact can be mitigated. For instance, one Middle Eastern study noted that married patients with vitiligo had longer marriage durations on average than controls, implying that those relationships remained stable. Researchers speculated that couples facing vitiligo sought counseling together or that spousal support helped improve the patient’s QoL.^49^ However, in more severe cases, vitiligo has been cited as a factor in divorce (likely when a spouse or in-laws hold strong stigmas or wrongly blame the affected individual). Culturally, educating partners about vitiligo (that it is not contagious and not the patient’s “fault,” among others) is vital. Some dermatologists encourage patients to bring their significant others to appointments so the physician can address any questions or misconceptions. This approach can strengthen the partner’s supportive role.

#### Impact on education and career

The social impact of vitiligo extends to school and workplace environments. In school, children with vitiligo may experience bullying or exclusion, affecting their academic participation. Some may skip school on days their patches are very visible or after a bullying incident. Teachers and school counselors should be involved to foster an inclusive atmosphere. In the workplace, adults with vitiligo may face subtle or overt discrimination. A review by Ezzedine et al. mentioned loss of job opportunities due to vitiligo and feeling less respected at work.^17^

A study in 2024 by Akl et al. used modeling to estimate overall burden and suggested that vitiligo contributes to reduced productivity and even workforce dropout in severe cases.^57^ Therefore, supporting patients in maintaining their educational and occupational ambitions is an important part of comprehensive care.

#### Coping mechanisms and social support

Despite these challenges, many patients with vitiligo develop coping strategies to maintain their social lives. Cosmetic camouflage is a widely used technique. High-quality concealers or self-tanning products can cover patches, and patients report increased confidence in social settings, without worrying about others’ reactions, when using such products.^36,61^ A study demonstrated that after cosmetic camouflage training, patients with vitiligo had significantly improved DLQI scores, indicating better QoL.^36^ Vitiligo management guidelines even recommend camouflage as an adjunct “therapy” to help patients function normally in social settings.^61^ Another important strategy is seeking support from others with vitiligo. Support groups (local or online) allow patients to share experiences and advice, reducing the sense of isolation.^44^ Additionally, cognitive-behavioral techniques (such as rationally reframing others’ reactions or building assertiveness to handle rude comments) can empower patients to face social situations more confidently.^44^

Families also play a critical role. Educating a patient’s family members about vitiligo can mitigate inadvertent negative remarks at home and help transform family into a solid support system. In pediatric cases, educating classmates can prevent bullying. For example, a simple classroom presentation about vitiligo by a school nurse reportedly transformed peers’ attitudes from teasing to empathy for a child with vitiligo.

In summary, the social impact of vitiligo is characterized by stigma (external and internal), altered social interactions, and challenges in relationships and life roles. The degree of social disability varies widely; some individuals, with confidence or support system, continue to engage fully in society, whereas others withdraw. Addressing the social impact requires both societal interventions (awareness campaigns and antidiscrimination efforts) and personal interventions (counseling, support networks, and coping skills training). Encouragingly, as general awareness improves and as more public figures speak openly about vitiligo, stigma may slowly diminish. Until then, support networks and coping strategies remain a lifeline for patients to reclaim their social lives.

### Economic impact

Beyond psychological and social challenges, vitiligo can also impose an economic burden on patients and healthcare systems. Although vitiligo is not life-threatening, it is a chronic condition that often requires long-term management; frequent healthcare visits; and, in some cases, costly treatments or ancillary products (such as cosmetics). Additionally, the psychosocial effects of vitiligo can indirectly impact productivity and work participation, resulting in economic consequences. Herein, we have discussed direct and indirect costs associated with vitiligo along with the evidence on patients’ willingness to pay (WTP) and actual expenditures.

Vitiligo is associated with substantial direct and indirect costs that are comparable to that of inflammatory dermatoses, largely mediated by psychosocial morbidity.

#### WTP for a cure

A useful measure to gauge the perceived burden of a disease is how much patients would hypothetically be willing to pay to cure it. Studies have demonstrated that patients with vitiligo report remarkably high WTP for a cure or highly effective treatment, often comparable to or higher than the WTP of patients with more symptomatic skin diseases.^41,56^ For instance, a survey found that vitiligo had the highest median WTP for a complete cure among several dermatologic conditions, even though, based on standardized QoL instruments (such the DLQI), vitiligo’s average impairment was only moderate.^56^ This apparent discrepancy, which is high WTP but relatively lower DLQI impact, underscores that traditional QoL questionnaires may underestimate the true burden of vitiligo (because they do not capture everything). In contrast, WTP captures the holistic burden that the patients experience.^56^ In this study, patients with greater QoL impairment (higher DLQI scores) were indeed willing to pay more, indicating that WTP is associated with perceived burden.^56^ These findings indicate that many patients value a vitiligo cure very highly, reflecting how life-altering they consider the condition. Some patients have reported that they would give up a significant portion of their income or savings to restore their skin’s pigment.^56^ This strong willingness for effective treatment is also evidence of the mental burden associated with vitiligo. It is not merely “cosmetic” as many people are willing to pay substantial amounts for a cure.

#### Direct medical costs

Vitiligo management can incur various medical expenses, particularly for patients undergoing treatment. Direct costs include doctor visits (dermatology consultations), treatment costs (topical medications, phototherapy sessions, and systemic drugs, among others), and laboratory tests (for associated conditions or therapy monitoring). Although vitiligo treatment options have historically been limited (many patients until recently had few effective options), the emergence of newer therapies (such as JAK inhibitors or home phototherapy units) may increase costs. A US insurance claims study found that annual healthcare costs for patients with vitiligo were higher than for matched individuals without vitiligo. This difference was partly driven by dermatology visits and therapies but also by more frequent mental health visits, as some patients sought psychotherapy or psychiatric care for vitiligo-related distress.^57^^,73^ An analysis suggested that the higher costs in patients with vitiligo “may partly be explained by a higher risk of mental health conditions,” which led to increased healthcare utilization (e.g. therapy sessions and antidepressants).^57^ Therefore, the psychological impact of vitiligo can directly translate to economic impact through additional medical care.

Regarding treatment costs, basic topical therapies (steroids and calcineurin inhibitors) are relatively inexpensive; however, their long-term use can further increase the cost. Phototherapy (narrow-band UVB) often requires 2–3 sessions per week for several months, and when administered in clinical setting, it includes not only procedural costs but also travel expenses and time off work. Some patients invest in home phototherapy units, which can be costly upfront. Emerging interventions like oral JAK inhibitors or melanocyte transplantation can be expensive (although these are not yet mainstream). A 2024 analysis by Ezzedine et al. estimated that patients with vitiligo in the US incurred on average around US$3400 more in annual vitiligo-related healthcare costs than individuals without vitiligo.^56^ Costs tended to rise with disease severity. Patients undergoing systemic treatments or combination therapies spent more than those managing with only cosmetics or observation.^73^ Additionally, because vitiligo is often accompanied by other autoimmune disorders, patients sometimes incur costs for managing those conditions (e.g. thyroid hormone replacement for autoimmune thyroiditis, which is common in vitiligo).^73^ A study reported that patients with vitiligo requiring systemic therapies (indicating more severe disease) had substantially higher medical expenses, and that coexistent autoimmune diseases such as thyroid disorder or diabetes further added to healthcare expenses.^73^

#### Non-medical and indirect costs

Beyond formal healthcare, patients with vitiligo often spend money on nonprescription items to manage their appearance and skin health. These include high-SPF sunscreens (used diligently to protect depigmented skin), protective clothing (such as UV-blocking garments), and cosmetic camouflage products (specialized makeup, self-tanners, and dyes),^73^ which are usually out-of-pocket. Although the cost of a single bottle of sunscreen or concealer may not appear significant, these expenses accumulate over time and can exceed the typical skincare spending of individuals without vitiligo. Particularly, image-conscious patients may invest in premium camouflage solutions or custom-blended makeup. There are even professional services for cosmetic tattooing or tinting of patches, which can be expensive and require periodic maintenance. All these non-drug costs were highlighted in a 2023 review by Naldi et al., which reported that patients with vitiligo have expenses “exceeding typical skincare expenditures” due to these additional needs.^55^

Furthermore, there are indirect costs, referring to lost productivity or income due to the condition. Vitiligo can indirectly lead to work or school absences; for example, an adult may take time off for thrice-weekly phototherapy sessions, or a child may miss school because of bullying-related distress. Over time, these missed opportunities (lower educational attainment and underemployment) can have economic repercussions. One specific indirect cost is time spent on care: patients devote time daily to applying makeup or sun protection and weekly during medical visits or UV sessions.^59^ This represents time not spent at work or on other activities, which economists sometimes quantify as a monetary loss. There is also evidence that work productivity may be reduced. A study reported that some patients with vitiligo are less productive at work, either due to psychological distraction (e.g. low self-confidence in public-facing tasks) or severe depression/anxiety, which may hinder their performance.^59^ In severe cases, individuals may avoid certain careers or opt for early retirement. Although vitiligo itself does not physically impair work ability, the associated cognitive and emotional burden can, in some individuals, translate to lower work engagement or even unemployment (e.g. individuals with severe vitiligo and comorbid depression may struggle to maintain a job).

Overall, although vitiligo is often not considered in economic terms, it imposes a considerable financial burden. For individual patients, this burden can be substantial, involving ongoing treatment costs (with uncertain benefit), spending on camouflage and sun protection, traveling to specialized centers, and even potential losses in work productivity or opportunities. At society level, considering the prevalence of vitiligo (up to 1%–2% globally), the aggregate cost (healthcare utilization along with lost productivity) is considerable. Nevertheless, vitiligo has historically received less attention in health economic research, likely because it is not fatal or acutely disabling. However, this is changing, with more studies advocating for resources to be allocated to vitiligo management corresponding with its impact.^57,59^ Recognizing the economic impact is also important for health policy. For example, insurance coverage of vitiligo treatments (such as phototherapy or new systemic drugs) can be justified by demonstrating QoL improvements and potentially avoiding downstream mental health costs.

In practical terms, physicians should be mindful of the financial burden on patients. Some individuals forego treatment or clinic visits due to cost, which can worsen outcomes. Therefore, discussing treatment goals is important. For instance, a patient with very limited means may prefer focusing on affordable cosmetic measures rather than expensive medical therapies with low likelihood of success. Conversely, a patient willing and able to invest resources should be guided toward treatments most likely to help. Adopting cost-effective management strategies (such as combining at-home phototherapy with periodic check-ins or using generic topicals when possible) can be helpful. Additionally, given the association between psychological distress and increased costs (via mental health comorbidities),^57^ effectively addressing the psychosocial aspect can indirectly reduce economic impact. For example, a well-supported patient may require fewer emergency mental health visits and demonstrate high productivity at work.

In summary, the economic impact of vitiligo underscores that its impact extends beyond the skin. Patients often incur direct costs for ongoing care and indirect costs through life adjustments. Further studies are warranted to precisely quantify this burden; however, existing evidence indicates that vitiligo is associated with considerable financial strain for many patients.^57^ Recognizing this burden should prompt healthcare providers and insurers to consider vitiligo as a condition worthy of coverage and support. For instance, covering effective treatments and support services may ultimately be cost-saving if they improve patients’ ability to function and remain productive.

This review has several limitations. As a narrative review, it does not follow the rigorous study selection and quantitative synthesis methods of systematic reviews or meta-analyses, which may introduce selection bias. The included literature spans multiple geographic and cultural contexts, potentially limiting the comparability of reported psychosocial outcomes. Additionally, heterogeneity in study design, QoL instruments, and outcome measures across studies makes direct comparison challenging.

## Conclusion

Vitiligo imposes a multidimensional burden that extends beyond visible skin changes. Its impact on QoL arises from the interplay among clinical features, psychological vulnerability, sociocultural context, and economic strain. Lesion visibility, stigma, and unpredictable disease progression contribute substantially to emotional distress, social isolation, and reduced self-esteem, often culminating in anxiety or depression. These consequences are particularly pronounced in women, younger patients, individuals with darker skin phototypes, and those experiencing cultural prejudice. Although existing QoL instruments offer useful insights, they do not fully capture this holistic burden. Stigma, coping behavior, and cultural variation remain insufficiently assessed. Equally important is ensuring timely access to highly effective therapies. Although current therapies are limited and under active development, they are capable of inducing rapid repigmentation and demonstrating durable disease control. Reducing lesion visibility not only improves clinical outcomes but also helps mitigate stigma, enhances self-esteem, and restores QoL. Comprehensive management must therefore integrate psychosocial support, patient education, and culturally sensitive counseling alongside medical treatment. Recognizing and addressing the hidden burden of vitiligo is essential for improving outcomes, guiding therapeutic decisions, and ultimately validating the lived experience of patients.

## Data Availability

All data used in this narrative review are derived from published studies available in the public domain. No new datasets were generated or analyzed for this manuscript.
